# Scalable Additive Construction of Arrayed Microstructures with Encoded Properties for Bioimaging

**DOI:** 10.3390/mi13091392

**Published:** 2022-08-25

**Authors:** Matthew DiSalvo, Belén Cortés-Llanos, Cody A. LaBelle, David M. Murdoch, Nancy L. Allbritton

**Affiliations:** 1 Microsystems and Nanotechnology Division, National Institute of Standards and Technology, Gaithersburg, MD 20899, USA; 2 Department of Bioengineering, University of Washington, Seattle, WA 98105, USA; 3 Department of Medicine, Duke University Medical Center, Durham, NC 27705, USA

**Keywords:** microarray analysis, microfabrication, bioimaging, single-cell analysis, barcoding

## Abstract

Microarrays are essential components of analytical instruments. The elements of microarrays may be imbued with additional functionalities and encodings using composite materials and structures, but traditional microfabrication methods present substantial barriers to fabrication, design, and scalability. In this work, a tool-free technique was reported to additively batch-construct micromolded, composite, and arrayed microstructures. The method required only a compatible carrier fluid to deposit a material onto a substrate with some topography. Permutations of this basic fabrication approach were leveraged to gain control over the volumes and positions of deposited materials within the microstructures. As a proof of concept, cell micro-carrier arrays were constructed to demonstrate a range of designs, compositions, functionalities, and applications for composite microstructures. This approach is envisioned to enable the fabrication of complex composite biological and synthetic microelements for biosensing, cellular analysis, and biochemical screening.

## 1. Introduction

Array-based technologies are ubiquitous across the fields of chemical and biological analysis. In particular, microarrays have fundamentally enabled massive scalability within small device footprints, which has granted the high technology value for research and commercial applications. Microarrays have been adopted as essential components for highly successful analytical instruments requiring high throughputs and multiplexing, including multi-omic sequencing, chemical and drug screening, and cytometry [1,2,3,4,5]. The implementations of microarrays can be categorized into two main strategies: planar (solid-state) or suspension (liquid-state) arrays [6,7,8]. Planar arrays situate analytes at indexed positions on a flat surface (typically a polymer or glass) and are thus amenable to imaging-based assays and combinatorial screening. However, planar arrays have a limited number of positions that limit assay scale. In contrast, suspension arrays associate analytes with microparticles, and these ensembles are then mixed together into one liquid vessel. Beneficially, suspension arrays are compatible with many high-throughput droplets- or microfluidic- based assays. A hybrid array format capable of combining aspects of both planar and suspension arrays would open new multiplexing possibilities.

Nevertheless, the sheer density of microfeatures within microarray technologies has created new opportunities—and new challenges–around the topic of information encoding [7,8,9]. Measurements on microarrays generally require encoded identifiers to link microelements, their associated analytes, and other components of the measurement system. For planar chemical microarrays, one classical approach is to selectively spot chemicals at known positions, or indices, on the array [10,11]. Using these known positions, measurement read-outs can then be assigned or registered to individual analyte spots. With the development of bioanalyses such as multi-omics that require a pipeline of multiple measurements performed on the same cell, more complex encoding is required to register measurements of the same analyte as it is processed. For example, in solution-based microarrays, encoding microbeads can be associated with the analyte as it transits different instruments, microarrays, or solutions. External microbeads have successfully encoded information within combinations of fluorophores or genetic sequences but can introduce practical challenges regarding their fabrication, microarray loading, and design of (bio)chemical association with analytes [6].

The next generation of encoded microarray fabrication would address microfabrication challenges to make it easier to create microarrays with many functions. Active or manipulatable microarrays can be highly desirable, for example, to enable reconfiguration and interoperation as both planar and solution formats. Broadly speaking, the production of such arrays is technically challenging since the ideal microfabrication method would be expected to achieve (1) microscale resolution, (2) broad material compatibility, and (3) the ability to customize individual array elements with different designs and materials. Photolithography is a well-established fabrication method used throughout industry and research and has proven successful for fabricating composite microarrays with high feature resolution and uniformity [12]. Photolithography can construct objects additively (material deposition via spin-coating, vapor deposition, or electroplating) as well as by subtraction (material removal by chemical development or lift-offs) [13]. Although many photoresists and metals are readily compatible with photolithography, using different materials (especially those sensitive to temperature, solvents, or light) generally requires optimizations and modifications to photolithographic methods that may not be feasible. Photolithography conventionally also requires access to cleanroom facilities and follows multistep time- and reagent-consuming protocols. Three-dimensional printing methods are compatible with some novel materials and have been used for low-cost fabrication of biosensor arrays, but currently still have limited speed and resolution for these applications [14]. Overall, the tradeoffs between accessibility, resolution, choice of materials, and speed for microarray fabrication are central barriers slowing the advancement of these microdevices.

This work reports a novel additive construction approach to the production of arrayed and encoded microstructures. Materials in liquids are deposited as building blocks using the phenomenon of discontinuous de-wetting, and these depositions are built upon over sequential steps to comprise organized structures. The approach easily manufactures customizable microarrays with variable microelement compositions and structures. Depending on the chosen materials, the deposition parameters, and the substrate topography, microarrays are producible with various element encodings and functions. As a proof-of-concept, this work develops microarrays of magnetically mobile, rigid, and optically transparent structures and shows how they can be reformatted from planar to suspension array styles. This study establishes the feasibility of using novel microelements as cellular micro-carriers in fluorescence bioimaging assays. As a final demonstration, fluorescent microparticles were patterned inside the microelements and were used as a microscope-readable barcode.

## 2. Materials and Methods

### 2.1. Materials

Carboxyl-functionalized 3 μm nominal diameter superparamagnetic maghemite microspheres (MC3001, Lot# 060116) were obtained from Ocean Nanotech (San Diego, CA, USA) (The full description of the procedures used in this paper requires the identification of certain commercial products and their suppliers. The inclusion of such information should in no way be construed as indicating that such products or suppliers are endorsed by the National Institute of Standards and Technology (NIST) or are recommended by NIST or that they are necessarily the best materials, instruments, or suppliers for the purposes described). Red, green, and blue (nominal excitation/emission at 365/405 nm, 520/525 nm, and 580/600 nm) polystyrene microspheres of 4 μm nominal diameter (Molecular Probes MultiSpeck M7901) were purchased from Invitrogen (Waltham, MA, USA). Gamma-butyrolactone (GBL) with ≥ 99% purity was used. Polysiloxane glass resin (GR650F) was obtained from Techneglas, Inc. (Perrysburg, OH, USA). The latent-HIV assay reagents were Roswell Park Memorial Institute (RPMI) 1640, Phenol-red free RPMI (11835030, + l-Glutamine) from ThermoFisher (Waltham, MA, USA), fetal bovine serum (FBS, GIBCO, 11835-030), penicillin/streptomycin (P/S, 10,000 units per 1 mL), 4-(2-hydroxyethyl)-1-piperazineethanesulfonic acid (HEPES) buffer (GIBCO, 15630-080), and Hoechst 33342 (H3570) from Invitrogen.

### 2.2. Microfabrication

**Figure 1:**
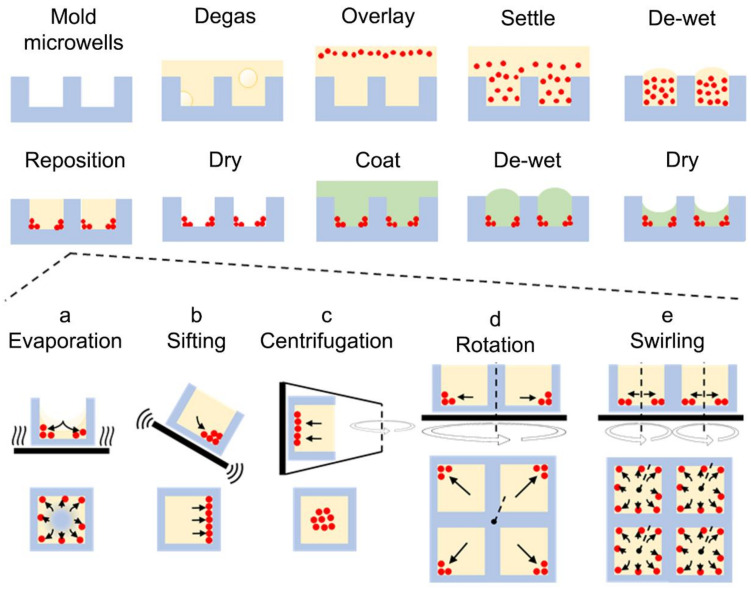
Overview of embedded microstructure arrays (“EMAs”) microfabrication. First, a polydimethylsiloxane (PDMS) microwell substrate is fabricated by soft lithography (details omitted). After wetting and degassing the substrate, microparticles in carrier fluid are loaded onto the substrate, and excess fluid is removed via discontinuous de-wetting. Microparticles are deposited to the base of the microwells via either (**a**) an evaporative process, moving the particles by surface tension; (**b**) mechanical sifting, allowing the particles to gravitate down a tilted substrate; (**c**) rotary centrifugation to force particles to the microwell base; (**d**) rotation around the plane of the substrate, moving particles with centripetal forces; or (**e**) swirling, where currents within microwells distribute particles. After microparticle deposition, the carrier fluid is removed by evaporation. Lastly, the arrays are dip-coated into a polymer solution embedding the deposited microparticles within the polymer and forming a molded composite microstructure. Blue: PDMS; Yellow: carrier fluid; Red: microparticles; Green: liquifiable polymer; Black arrows: direction of microparticle motion. The bottom rows of panels (**a**–**e**) represent the top-down perspective of the microwells.

The microfabrication process is illustrated in Figure 1. Polydimethylsiloxane (PDMS) microwell array substrates were molded from photoresist-on-glass templates and temporarily laminated to a glass backing using water-soluble poly(acrylic acid) glue as previously described (Figure 1) [15,16,17]. Carrier fluid (water, ethanol, or GBL, total volume of 155 μL per 1 cm^2^ of array surface area) was added to the microarray and was retained in place by a sealed PDMS chamber. The carrier fluid was degassed for 1 min by a vacuum pump. A separate volume of carrier fluid (92 μL per 1 cm^2^) was prepared containing microparticles (375 μg mL^−1^ unless otherwise noted) and was mixed by 2 min sonication followed by vortexing. This mixture was added on top of the carrier-fluid-wetted microarray with two rounds of up and down micropipetting. After settling for 1 min, the supernatant fluid was removed from the microarray by pipetting (Figure 1). Then, optionally, the microarray was: heated at 95 °C to evaporate the carrier fluid and move the particles (Figure 1a); sifted by 100 Hz agitation for 30 min on a vibration table at 26° tilt angle (Figure 1b); centrifuged with a relative centrifugal force of 7.8 kN kg^−1^ for 2 min in a swinging-bucket centrifuge (Figure 1c); or rotated by a spin-coater for 10 s at 52 rad s^−1^ followed by 40 s at 420 rad s^−1^ (Figure 1d). Lastly, in all cases, the array was heated at 95 °C for up to 1 h to evaporate any remnant carrier fluid. To embed dried microparticles that were deposited into microarrays, the microarrays were dip-coated as previously reported [15,16] using a solution (mass fraction of 20%) of either polystyrene in GBL or glass resin in ethanol and dried overnight at 95 °C (Figure 1).

A modification of the microfabrication protocol was used, referred to as the “swirling” approach. Here, the same procedures were followed, but the initial γ-Fe_2_O_4_ microsphere concentration was halved (to 0.1875 mg mL^−1^), and prior to the 1 min setting period, the microarray was swirled at 8.5 rad s^−1^ for 3 min by an orbital shaker (Figure 1e). After removing the supernatant, the array was swirled at 17 rad s^−1^ on the orbital shaker for 90 min at 20 °C.

### 2.3. Microstructure Characterization

Microparticle loading distributions were characterized from fluorescence microscopy images that were acquired by a previously described fluorescence microscopy scanner composed of a motorized epifluorescence microscope using a CMOS camera with a pixel size of 6.5 μm (referred to hereafter as the “scanning fluorescence microscope”) configured with a semi-apochromatic objective (0.13 numerical aperture [NA], 4× objective magnification) and 562/624 nm excitation/emission [17]. Fluorescence areas were quantified from the images by a previously described numerical computing analysis pipeline [18]. Microstructure morphology and chemical composition were studied using a scanning electron microscope (SEM) equipped with an energy dispersive spectroscopy (EDS) system. Three-dimensional structural composition of microelements was obtained by laser scanning confocal fluorescence microscope and air immersion semi-apochromatic objective (0.5 NA, 20× magnification) for material autofluorescence with excitation/emission at 500/540 nm or 570/670 nm. Imaging of microstructures released from the substrate was performed with an inverted epifluorescence microscope equipped with a CCD camera with a pixel size of 6.45 μm (referred to hereafter as the “non-motorized fluorescence microscope”). Two systems were used to characterize the magnetic transfer efficiencies: a previously described motorized 3-axis magnetic pick-and-place system and an objective-mounted microneedle [19,20]. These systems tested the ability to attract magnetic microelements (100 μm × 100 μm × 60 μm; width × height × depth) to a magnetic wand and transfer them between two liquid reservoirs.

### 2.4. Latent-HIV Assay

Latent HIV Jurkat cells (J-Lat 6.3, NIH HIV Reagent Program) with a GFP reporter gene were seeded onto microarrays with microwell geometry of 100 μm × 100 μm × 60 μm (width × height × depth). Phenol-red free RPMI was supplemented with FBS (10%), P/S (1%), and HEPES (20 mmol L^−1^). Cells were stained with Hoechst 33,342 (1 mg mL^−1^), and two latency reversal agents were added to stimulate the reactivation: phorbol myristate acetate (80 nmol L^−1^) and ionomycin (0.5 μmol L^−1^). The arrays were scanned by an automated platform for 24 h at 8 h intervals in brightfield, blue fluorescence, and green fluorescence channels [19,21]. Microelements containing single cells were identified, and their respective fluorescence emission signals in the blue and green channels were compared. To measure fluorescence, the Hoechst signal was used to locate the center of each cell. Next, a circular mask (20 μm diameter) was created from the centroid of each cell mask to measure integrated fluorescence in the blue and green fluorescence channels. The integrated fluorescence measurements were adjusted by calculating the average background of the cell’s respective micro-carrier and subtracting the value from the integrated fluorescence within the cell mask.

### 2.5. Barcoding

To fabricate embedded microstructure arrays (EMAs) barcoded by fluorescent polystyrene microspheres, water was used instead of GBL as the carrier fluid to prevent the dissolution of the microspheres. Polysiloxane glass resin in ethanol was used as the embedding polymer. The barcoded structures were formed within 500 μm × 500 μm × 200 μm (width × height × depth) microwells. Planar microarrays were imaged by the scanning fluorescence microscope configured with a semi-apochromatic objective (0.3 NA, 10× objective magnification, and fluorescence filter sets with excitation/emission at 350/460 nm excitation/emission, 482/536 nm, and 562/624 nm. Released microarray elements in suspension were imaged by the non-motorized fluorescence microscope configured with a flat-field fluorite objective (0.5 NA, 20× objective magnification).

Cropped images of fluorescent microstructures originating from different microscopes were aligned using similarity affine transformations found from multimodal intensity-based registration [22]. Cropped images obtained from the same microscope were aligned using rigid transformations found from cross-correlation [23]. All aligned and processed images shared a common pixel size of 0.625 μm. A tophat filter was then used to subtract the background from aligned images using a size of 32 μm, or 8 bead diameters. To equalize the intensities of images, the processed images were manually thresholded with 8 global intensity levels per color channel. To reduce sensitivity to small alignment shifts, the thresholded images were dilated by 6 μm or 1.5 bead diameters. Finally, the images were linearized to form barcodes (3 colors × N pixels). For computational speed, only the top N = 50,000 pixels that were most frequently occupied by microbeads were used. Barcodes were compared using the Pearson correlation coefficient.

### 2.6. Statistical Analysis

Data are presented as the mean ± standard deviation unless otherwise noted. Dispersion in the deposition of microbeads was calculated as the robust coefficient of variation (*rCV*) [24].

(1) $$rCV=\frac{k\cdot median\left(\left|X_{i}-median\left(X\right)\right|\right)}{median\left(X\right)}$$

The scale factor *k* was given by

(2) $$k=1/\left(\Phi^{-1}\left(3/4\right)\right)\approx 1.4826$$

where Φ denotes the cumulative distribution function of the standard normal distribution. The standard error of *rCV* was estimated via bootstrap with 1000 data samples taken with replacement. All computational and statistical analyses were performed using MATLAB (MATLAB 2019b, The MathWorks, Inc., Natick, MA, USA).

## 3. Results and Discussion

### 3.1. Carrier-Fluid-Mediated Deposition of Microparticles

In microstructure fabrication, discontinuous de-wetting, or the complete de-wetting of a surface by a solution moving over a material with high surface energy, has been used to coat substrates [25,26]. We envisioned a microfabrication approach that utilized de-wetting fluid to carry and position particles along a surface. If this surface contained wells, particles could be specifically deposited within them as the carrier fluid de-wets away. To test this idea, a substrate with uneven topography was overlaid with a carrier fluid suspending particles (Figure 1). The substrate was composed of PDMS, a hydrophobic thermoelastomer material whose topography was readily altered by soft-lithographic molding. As a preliminary test, the substrate was patterned with large microwells (500 μm × 500 μm × 200 μm width × height × depth) and overlaid with GBL—a compatible solvent with PDMS—suspending 3 μm diameter γ-Fe_2_O_4_ (maghemite) microspheres (inert to GBL). As the GBL evaporated, a concave meniscus was formed, whose depth lowered over time (Figure S1, Supporting Information). At a critical point, the meniscus contacted the base of the microwell, after which the GBL discontinuously de-wetted from the base of the well along a radially processing front. The microspheres were carried along the front, and after complete evaporation of the GBL carrier fluid, they were patterned within the microwell.

**Figure 2:**
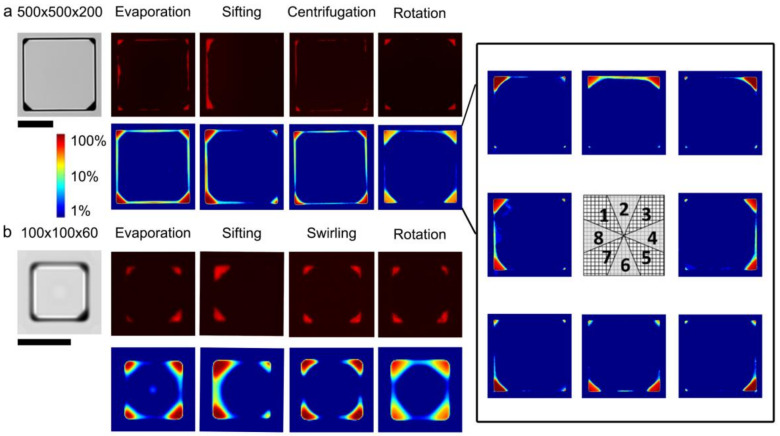
Analysis of microparticle deposition patterns. γ-Fe_2_O_4_ microspheres were deposited into large microwells (**a**); scale bar = 250 μm and small microwells (**b**); scale bar =100 μm. Top rows: representative brightfield and fluorescence microscopy images; Bottom rows: Probability density maps colored with a logscale colormap indicating the pixel-wise likelihood of particle occupancy, processed from over at least 1200 or 19,000 microwells for large and small microwells, respectively. Right inset: probability maps extracted along octants of a microarray (center schematic, one representative map is shown from each octant) illustrating an angular gradient for rotationally deposited particles.

To explore potential influences on the deposition of microparticles, the particle settling phenomena were analyzed from fluorescence microscopy of microwells. To provide a relatively large sample size of microwells, the microwells were arrayed on the substrate at up to 2500 microwells per 1 cm^2^. Deposition patterns varied by fabrication approach and microwell scale (Figure 2; see Materials and Methods for fabrication details). Simply evaporating the solvent after loading particles in the microwells allowed the surface tension to move particles uniformly towards the microwell perimeter and four corners. It was found that swirling the microarray with a circular motion during this drying appeared to improve the consistency of this deposition pattern. Thus, several options were tested in an attempt to further influence the motion of particles and alter their deposition locations after being dried. For example, it was observed that tilting and vibrating the microwells allowed the liquid-borne particles to settle to one side of the microwells and remain there throughout the drying process. Alternatively, rotating the microwells on a spin-coater pushed liquid-borne particles away from the center of rotation, consistent with the direction of centripetal force. By centering the microarray on the revolving surface, a rotational gradient in deposition patterns was produced. For all methods, particles were moved away from the middle of the microwell during drying. Centrifugation of the microarrays did not prevent this motion. When the microwell volume was reduced by a factor of 80, from 500 μm × 500 μm × 200 μm down to 100 μm × 100 μm × 60 μm (width × height × depth), the particles were more strongly and unpredictably moved to the microwell corners, suggesting stronger surface tension effects (Figure 2b). These results are evidence that batch fabrication achieved a selection of uniform and variable microparticle deposition patterns.

The relationship between microparticle quantity and deposition pattern variability was studied to explore an additional level of control on the microfabrication process. A baseline level in microparticle loading variability was first established in the smaller 100 μm × 100 μm × 60 μm (width× height × depth) microwells with a particle concentration of 188 μg mL^−1^ and a modification to the evaporative fabrication approach that used swirling to redistribute the particles. The baseline percent fluorescence area relative to the microwell cross-sectional area was 4.0% ± 1.0%. The variability in fluorescence areas across this microarray was characterized by the robust coefficient of variation (*rCV*) of 22.2%, with an estimated *rCV* standard error (*SE_rCV_*) of 0.2%. Without swirling, the fluorescence area was 3.7% ± 2.0% (*rCV* = 45.1%, *SE_rCV_* = 0.4%). Next, in the swirling condition, the microparticle loading concentration was lowered to 47 μg mL^−1^ or raised to 750 μg mL^−1^. The respective fluorescence areas were 0.4% ± 1.4% (*rCV* = 60.4%, *SE_rCV_* = 2.6%) and 13.3% ± 2.8% (*rCV* = 16.4%, *SE_rCV_* = 0.2%) (Figure S2, Supporting Information). The inverse relationship between dosage and dosage variability was consistent with the behavior of shot noise resulting from microparticle settling via a Poisson process. These results demonstrated the feasibility of controlling microparticle deposition dosage and pointed towards effective methods for influencing the consistency of deposition patterns in microwells.

### 3.2. Embedded Microstructure Arrays for Magnetic Conversion from Planar to Suspension Formats

**Figure 3:**
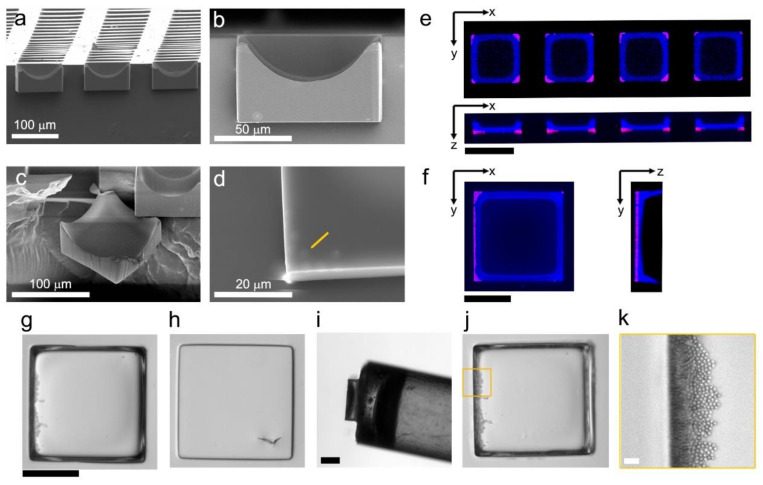
Morphological, structural, and magnetic characterization of fabricated EMAs. (**a**–**d**) SEM images showing cross-sections of an EMA with magnetic microparticles embedded in polystyrene sitting within 100 μm × 100 μm × 60 μm (width × height × depth) microwells. The magnetic microspheres (see yellow arrow) are faintly visible within SEM images due to charging effects. (**e**,**f**) Confocal fluorescence microscopy composite images of two sizes of magnetic polystyrene (MPS) embedded microstructure (EM) elements. Microspheres (red) are shown as maximum intensity projections; polystyrene (blue) is shown as median intensity projections. (**e**) Scale bar: 100 μm. (**f**) Scale bar: 250 μm. (**g**–**k**) Brightfield microscopy images illustrating the magnetic manipulation process: (**g**) representative MPSEM element; (**h**) microwell after microneedle ejection of the MPS EM element; (**i**) magnetic wand holding the released MPS EM element; (**j**) MPS EM element after deposition into a liquid filled well plate, with a magnified view (**k**, yellow square) showing the embedded magnetic microspheres visible through the transparent polystyrene. Scale bars: (**g**–**j**): 250 μm; (**k**): 20 μm. Microstructures in panels (**a**–**e**), (**f**), and (**g**–**k**) were prepared with the swirling method and sifting method, respectively.

We observed that the deposition of increasing amounts of material within microwells could form microstructures. To add larger amounts of material at once, fluids carrying dissolved polymer were deposited in the same fashion as the microparticles. After evaporation of the solvent, the polymer filled the microwell bottoms and encased previously deposited material to form solid, monolithic microstructures. These microstructures were formed with γ-Fe_2_O_4_ microparticles, GBL, and solution with a mass fraction of 20% polystyrene in GBL to create solid γ-Fe_2_O_4_ -and-polystyrene structures in PDMS microwell arrays. The magnetic particles in polystyrene EMAs were characterized to (1) identify the structure of the array elements and (2) confirm the presence and position of microspheres embedded within the elements. First, cross-section SEM images of magnetic polystyrene EMAs (“MPS EMAs”) revealed the microelement shape: rectilinear at the base with a concave upper surface, validating that the elements replicated the shape of the underlying microwells (Figure 3a–d). The microspheres were located along the bottom corners of the microstructures and presented with sizes consistent with their nominal diameter of 3 μm (see arrow on Figure 3d). For further validation, the elemental composition of MPS EMAs was spatially evaluated using EDS line scans (Figure S3a–c, Supporting Information). The relative elemental mass concentrations obtained from the spectra were C: 78.4% ± 4.6%, O: 11.7% ± 2.6%, Si: 6.3% ± 2.3%, and Fe: 2.4% ± 0.4% (N = 3 microelements). Fe within EDS spectra manifested a double Fe peak (0.70 keV and 6.43 keV) and O (0.53 keV) corresponding to γ-Fe_2_O_4_ [27,28,29]. A control polystyrene EMA (Figure S3d–f, Supporting Information) that was fabricated without magnetic microspheres presented no detectable Fe since it was lower than the background detection (2–3 counts); detectable elemental masses were C: 83.1% ± 0.4%, O: 10.3% ± 0.2%, and Si: 6.5% ± 0.2% (N = 3 microelements). The above EDS results corroborated the composition of MPS EMAs in a PDMS substrate. Supporting these results, confocal microscopy images also indicated that the microspheres were located at the bottom surface of the microstructures (Figure 3e,f).

The magnetic activity of MPS EMAs allowed them to be reconfigured by manipulation under magnetic fields. Using a previously developed system [19], the microstructures were ejected from the array by microneedles and collected to the tip of a magnetic wand (Figure 3g). For a preliminary test, the wand was moved by hand to collect MPS EMs fabricated by evaporating, sifting, rotational, and swirling conditions (Figure S4, Supporting Information). The collection efficiency (percent of microstructures deposited into the secondary vessel) for these respective conditions was 70% (evaporation), 35% (sifting), 30% (rotation), and 100% (swirling) (N = 30 microstructures per condition) for an aggregate efficiency of 58.8% ± 32.8%. Since the swirling condition more evenly distributed the γ-Fe_2_O_4_ particles, this condition was selected for a follow-up collection test using an automated 3-axis motorized gantry to transfer the microstructures to a liquid-containing well-plate. These automated collections had efficiencies of 95.6% ± 1.0% (N = 3 arrays, 60 microelements per array). These results demonstrate the feasibility of converting MPS EMAs between planar- and solution-based formats and suggested influences for particle deposition patterns and magnetization methods on the stability of magnetic attractions.

### 3.3. EMAs as Microcarriers for Bioimaging

**Figure 4:**
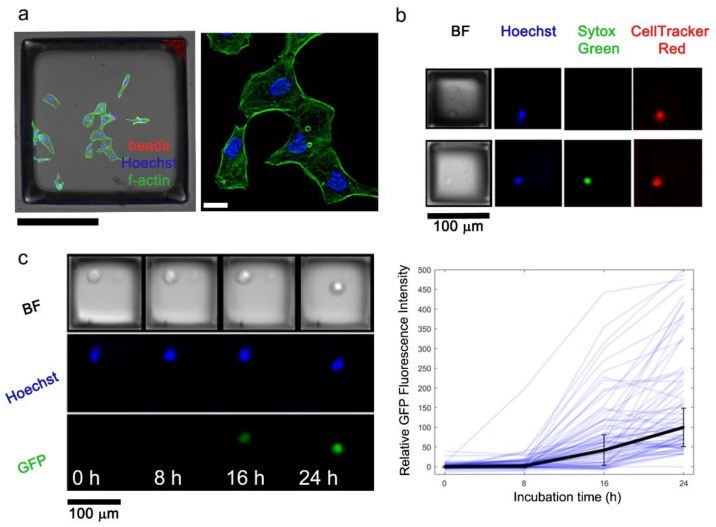
Technical demonstration of bioimaging assay applications on magnetic polystyrene EMAs. (**a**) Composite brightfield and fluorescence confocal microscopy composite image of fixed adherent A-431 cells (left); magnified view of a maximum intensity projection with subcellular fluorescence resolution (right). Black scale bar: 250 μm; white scale bar: 20 μm. (**b**) Representative widefield microscopy images of non-adherent Jurkat cells from fluorescence viability assays. (**c**) Representative widefield microscopy images (left) and all N = 257 activated single-cell fluorescence profiles (right) showing the increasing fluorescence of a GFP reporter in non-adherent J-Lat 6.3 cells. The solid black trace represents the median values; error bars are median absolute deviations. Microstructures in panels (**a**–**c**) were prepared with the rotational method, evaporative method, and swirling method, respectively.

Bioimaging assays impose several requirements on sample substrates: high transmission, low autofluorescence, and (for compatibility with objective working distances) substrate thinness. MPS EMAs constructed on 300 μm thin PDMS substrates exhibited high transmittance for UV-vis wavelengths that matched the transmittance of polystyrene EMAs, and the MPS arrays appeared clear in color to the eye (Figure S5, Supporting Information). To challenge bioimaging on the MPS EMAs, adherent A-431 cells were cultured and fixed on the MPS elements, and the feasibility of subcellular imaging was tested (Figure 4a). For this test, the MPS elements were molded within larger 500 μm × 500 μm microwells to produce flattened elements as described above. Confocal microscopy of A-431 cells adhered to these “micro-coverslips” revealed clearly visible actin filaments and nuclei above the background levels. Fluorescence background intensities were elevated near the γ-Fe_2_O_4_ microspheres, but since these were located at the micro-coverslip perimeter, this background did not contribute to images of cells.

Although an exhaustive demonstration of bioimaging assays was outside the scope of the work, we selected two relevant applications for EMAs. Cell viability and temporal protein expression were chosen because these assays are core tools for biological studies. Jurkat cells cultured on MPS EMAs were assessed using fluorescent labels to demonstrate a viability assay. Quantification of Hoechst, Sytox Green, and CellTracker Red biomarkers using wide-field microscopy showed 84.2% ± 4.5% CellTracker+ cells and 7.0% ± 0.5% Sytox Green+ non-viable cells, relative to the total Hoechst+ cell number (N = 12614 Hoechst+ total cell count summed over 3 microarrays) (Figure 4b). Temporal protein expression was performed on an HIV latency cell line with an inducible GFP reporter gene (Figure 4c). Each microculture element on the planar MPS EMAs was spatially indexed to facilitate tracking the expression levels of these cells over time by an automated fluorescence microscope. The responses of over 4900 total single cells were tracked over the course of 24 h of chemical stimulation between three separate experimental trials. Activating cells reached 50% of their final fluorescence response (defined as the median response at 24 h) at 15.3 h ± 4.6 h, demonstrating a characterization of the activation kinetics. Overall, the assay results supported the technological feasibility of performing fluorescence bioimaging assays using EMAs.

### 3.4. Stochastic Barcoding

Encoding strategies are needed to associate cells (and their contents) to specific cell-carrier elements to enable more complex bioassays. Traditional barcodes are quantized patterns that can be deterministically printed to allow for rapid and precise identification of a sample. We observed that the patterns of microspheres in MPS EMAs appeared slightly different for each microelement due to the random nature of particle depositions. We hypothesized that microsphere deposition patterns might be sufficiently distinct to encode the identity of the microstructure. Microscopy imaging could decode the particle patterns, although the quantization level of the code would be highly variable, depending on (1) the microbead size, (2) optical resolution and depth of focus, (3) camera pixel size, and (4) the number of bins used to represent image intensity. A limitation of this strategy is that it may be possible to observe identical particle deposition patterns. However, by increasing the dimensionality and variability of the patterns, the probability of identical codes can be reduced.

**Figure 5:**
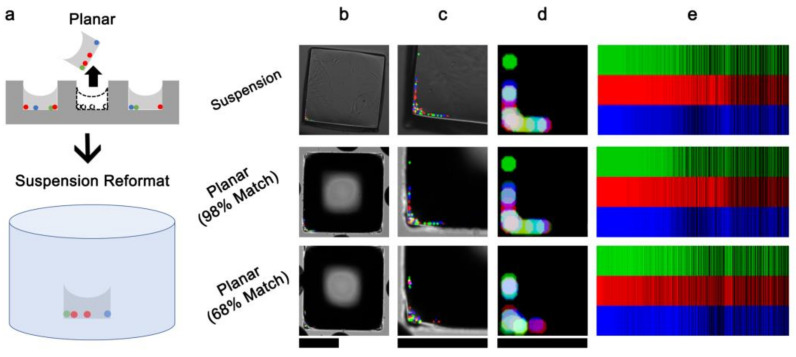
EM barcoding using embedded fluorescent microspheres. (**a**) Schematic of reformatting planar EMAs to suspension EMAs. (**b**–**e**) Representative barcoding results for an EM imaged in suspension and planar formats (top and middle rows), in contrast to a distinct non-matching EM (bottom row). (**b**) Composite widefield microscopy image of the EM element, scale bar: 250 μm; (**c**) magnified view of the encoding microbeads, scale bar: 200 μm; (**d**) registered and processed composite image, scale bar: 200 μm; (**e**) tricolor barcodes (for clarity, only the first 1500 codes are shown). Microstructures were prepared using the rotational method.

To test the feasibility of EMA “barcoding”, we replaced the magnetic microspheres with a mixture of red, green, and blue fluorescent polystyrene microspheres, which were deposited evaporatively along the periphery of microwells and then embedded by a glass resin microstructure (Figure 5). A fluorescent EMA was fabricated a bead fluorescence area of 0.36% ± 0.70% (*rCV* = 132.0%, *SE_rCV_* = 4.6%) relative to the microwell cross-sectional area; this high variability was chosen to ensure distinctness between deposition patterns. Using microscopy, automated image registration, and image analysis, 600 kbit barcode identifiers (50,000 codes with 8 intensity levels × 3 channels per microelement) encoding spectral and spatial information were obtained (see Materials and Methods). Then, N = 3 microelements were ejected from the array, reformatted as a suspension microarray in microwell plates, and re-imaged on a different microscope and objective (Figure 5b). The microelement identities were correctly matched to the original barcodes against a pool of 238 candidate barcodes according to the maximum-ranking correlation coefficient. The correlation coefficients were 90.7% ± 7.7% for the top-ranking matches; in contrast, the correlation coefficients for the top-ranking matches; in contrast, the correlation coefficients for the second closest matches were 71.7% ± 2.6%. These results demonstrated that microscopy-based barcoding was able to track EMs between planar and suspension formats.

For a larger-scale test, we used barcoding to track N = 101 array elements during the microfabrication process. Specifically, the barcode was encoded after the microsphere deposition but before embedment in resin. After resin embedment, the elements were re-imaged, and 94.1% were identifiable by their top-ranking barcode. The correlation coefficients were 81.9% ± 11.3% for the top-ranking matches; the correlation coefficients for the second closest matches were 73.5% ± 5.0%. These results highlighted the feasibility and robustness of spatial and spectral barcoding using EMAs and microscopy imaging.

## 4. Conclusions

The fabrication methods introduced in this work represented an additive construction approach that stochastically deposited liquid-borne microparticles and polymers. Many patterns of deposited materials were achieved without the need for individual design or tooling, allowing the method to be fundamentally compatible with the batch production of elements to form EMAs. The customization of array elements was possible holistically across the array or alternatively along gradients. The minimal fabrication requirements for EMAs (essentially, the chosen microparticles and embedding materials must be temporarily liquid-borne) open opportunities to create novel composite microstructures. The abundance of currently available functional microparticles enables our fabrication approach to access and use novel materials that are incompatible with existing photolithographic or printing methods. The demonstrated deposition approach lacks the deterministic control over material placement that is found in other methods, and its resolution is limited by the size of deposited microparticles. However, these tradeoffs were balanced with large advantages in the speed, accessibility, and simplicity of fabrication and allowed the choice between different materials without further optimizations. For example, entirely different deposition patterns (including gradients) were rapidly iterated because they were produced without needing to explicitly draw lithographic photomasks or 3D printing files for each design.

We envision that these fabrication techniques can be creatively applied to make arrayed bioimaging analyzers. For example, combinatorial patterning of chemical-doped microparticles within porous cell microcarriers would produce arrays with the potential to perform pharmacology, chemotaxis, or cell invasion assays. The roles of the surface chemistries of the materials and the topography of the substrate remain an interesting open question. These properties may be utilized to refine and expand the “toolbox” of available deposition patterns, i.e., by guiding discontinuous de-wetting behavior or molding different bulk microstructure shapes. Further characterizations of barcoding capacity and decoding accuracy will be needed to optimize deposited particle barcodes for use in assays with larger sample sizes. Nevertheless, using multiple bead colors, intensities, and patterns is a path to maximizing the information density and robustness of identifiers for different types of optical decoders. Although many advances are needed to bridge the gap between planar and suspension microarray formats for multiplexed bioassays, the demonstrations of (1) selective reformatting of elements from planar to suspension microarray formats in MPS EMAs, (2) compatibility of EMAs with microscopy for bioimaging, and (3) internal encoding of EMAs represent substantial progress towards this goal.

## Data Availability

Data available upon request.

## Supplementary Materials

The following supporting information can be downloaded at: https://www.mdpi.com/article/10.3390/mi13091392/s1, Supporting Experimental Information, Figure S1: dynamics of evaporative particle deposition, Figure S2: quantitative analysis of microparticle deposition patterns, Figure S3: chemical composition of embedded microstructures, Figure S4: representative brightfield images of MPS EMs, Figure S5: characterization of optical transparency.
