# Supplementary material for: Scalable Additive Construction of Arrayed Microstructures with Encoded Properties for Bioimaging
Source: Micromachines (Basel). 2022 Aug 25;13(9):1392. doi: 10.3390/mi13091392 (PMC9500771; doi:10.3390/mi13091392)
Supplement: Supplementary file 1 [file micromachines-13-01392-s001.zip › micromachines-1867729-supplementary.pdf]

Supporting Information

Scalable Additive Construction of Arrayed Microstructures with Encoded Properties for Bioimaging

Matthew DiSalvo [1,†], Belén Cortés-Llanos [2,3,†], Cody A. LaBelle [2], David M. Murdoch [3] and Nancy L. Allbritton [2,*]

1 Microsystems and Nanotechnology Division, National Institute of Standards and Technology, Gaithersburg, MD 20899, USA
2 Department of Bioengineering, University of Washington, Seattle, WA 98105, USA
3 Department of Medicine, Duke University Medical Center, Durham, NC 27705, USA
* Correspondence: nlallbr@uw.edu
† These authors contributed equally to this work.

Supporting Experimental Information

Bioreagents: The cells used for conventional actin and viability bioimaging assays were A-431 epidermoid carcinoma cells and Jurkat T-lymphoblasts (clone E6-1). The assay reagents were Dulbecco's Modified Eagle Medium (DMEM), Phosphate-buffered Saline (PBS), Roswell Park Memorial Institute (RPMI) 1640, fetal bovine serum (FBS, GIBCO, 11835030), penicillin/streptomycin (P/S, 10,000 units per 1 mL), CellTracker Deep Red (ThermoFisher, C34565), Sytox Green (ThermoFisher, S7020), ActinGreen 488 ReadyProbes Reagent (ThermoFisher Scientific, R37110), Hoechst 33342 (Invitrogen H3570), and Triton-X-100 surfactant (GIBCO, 15250-061).

Bioimaging assays: For all bioimaging experiments, the PDMS microarrays were delaminated from the glass backings to produce a freestanding membrane which was suspended under a media-filled culture chamber. Prior to cell culture, the microarrays were plasma-treated for 5 min and sterilized with 70% ethanol. The density of cell suspensions was measured by a hemocytometer. Then, for each microarray, a volume of cell suspension was prepared with a total cell count equal to the quantity of microcarriers on the array and was pipetted onto microarrays to seed the arrayed cultures. The microarray samples were imaged using inverted microscopes equipped with software autofocusing to maintain a consistent focal level, as previously described [1]. The UV-visible absorption of the devices was measured using a plate reader. Actin filament assays were performed using a laser scanning confocal fluorescence microscope and air immersion semi-apochromatic objective (0.5 NA, 20× magnification) with excitation/emission at 405/470 nm, 488/540 nm, and 641/670 nm. Viability and gene expression assays were conducted by imaging using

a customized, motorized, and incubated widefield microscope with readouts extracted by image analysis as reported previously [2,3].

Actin filament assay. A-431 epidermoid carcinoma cells were seeded onto microarrays with 500 ◎m × 500 ◎m × 200 ◎m (width × height × depth) microwell geometry and cultured for 48 h in DMEM with 10 % FBS and 1 % P/S. The cells were then fixed with 4 % paraformaldehyde for 15 min and permeabilized with 0.5 % surfactant in PBS for 20 min. The actin filaments of the cells and the cell nuclei were co-stained with ActinGreen 488 and Hoechst 33342 (2 μg mL^{-1} in PBS) for 30 min.

Viability assay. Jurkat cells were cultured in RPMI 1640 complete media (10 % FBS and 1 % P/S) and stained with 1 μg mL^{-1} Hoechst 33342 and 1 μmol L^{-1} CellTracker Red for 30 min. The Jurkat cells were centrifugally rinsed and added to 3 mL of cell media with Sytox Green at 50 nmol L^{-1} and seeded onto microarrays with a microwell geometry of 100 μm × 100 μm × 200 μm (width × height × depth). The arrays were then imaged, and using a circular analysis mask centered on each cell, the integrated fluorescence intensity in each channel was measured after subtracting the background autofluorescence.

Supporting Figures

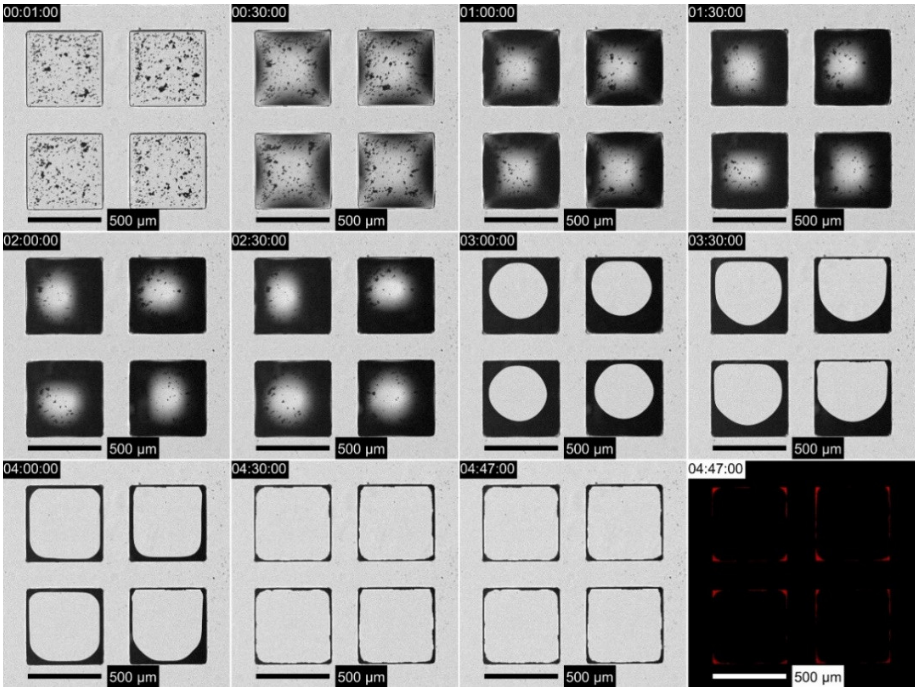

Figure S1. Dynamics of evaporative particle deposition. Brightfield microscopy images are shown at 30 min intervals for PDMS microwells loaded with maghemite microparticles in GBL carrier fluid. At room temperature, the carrier fluid slowly evaporates, and the dewetting fluid carries microparticles towards the microwell edges. The final image in the montage is a fluorescence microscopy image of the deposited microspheres.

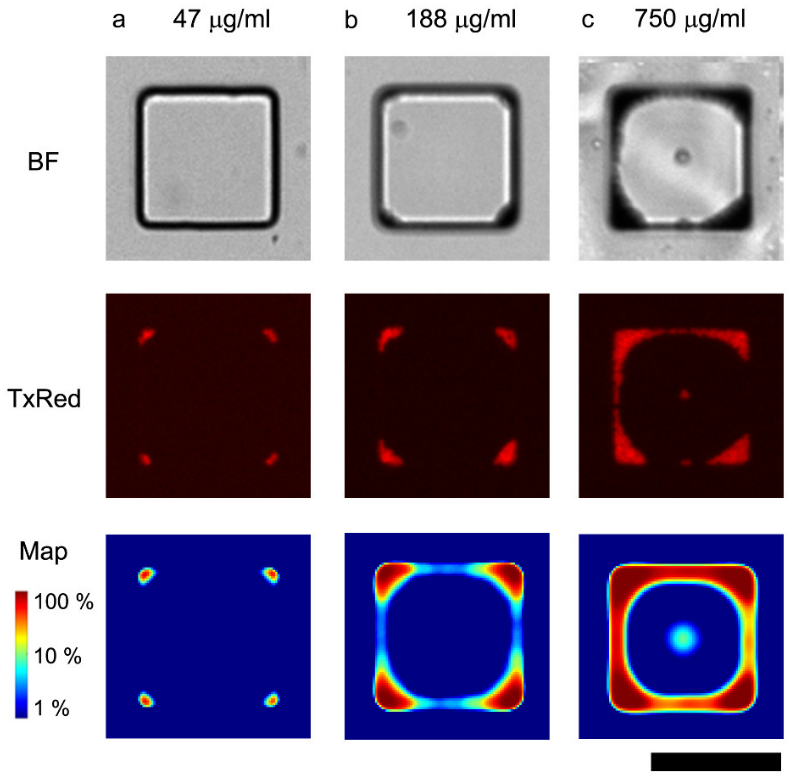

Figure S2. Quantitative analysis of microparticle deposition patterns with varying bead loading concentrations when using the swirling preparation method. Top row: representative brightfield microscopy images; Middle row: representative fluorescence microscopy images; Bottom row: Probability density maps, colored with a logscale colormap, indicate the pixel-wise likelihood of particle occupancy and were processed from over 19,000 microwells per condition. Scale bar: 100 µm.

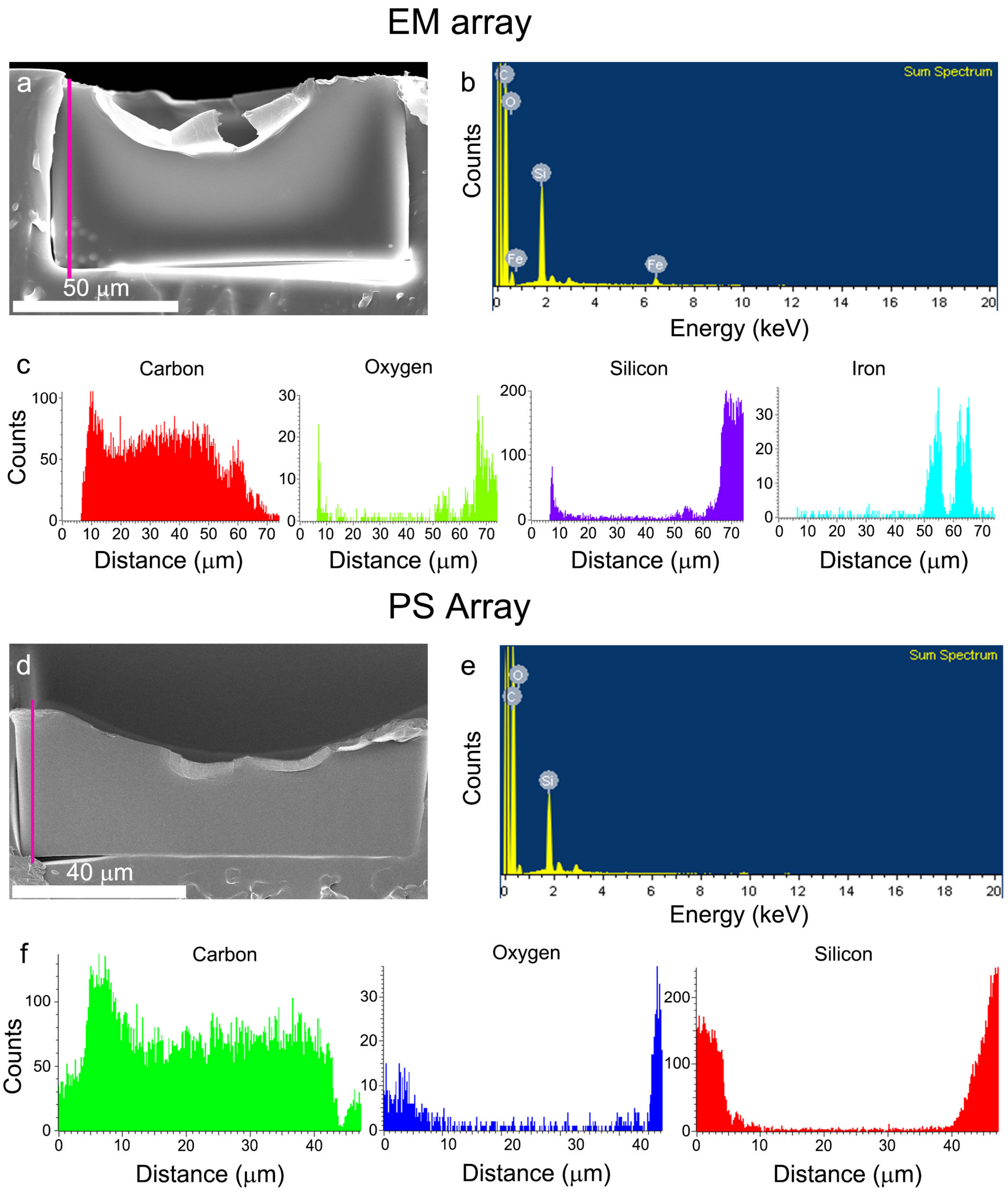

Figure S3. Chemical composition of embedded microstructures (EMs) by EDS-SEM. (**a**)-(**c**): Analysis of a maghemite-polystyrene EM prepared with the swirling method: (**a**) SEM cross-section image with EDS line trace overlaid in magenta, (**b**) EDS sum spectra, and (**c**) EDS spectra separated by element elements. Signals for Fe were observed for EDS line scans passing across a maghemite microbead. (**d**)-(**f**): Same analysis from a control polystyrene EM fabricated without embedded maghemite microspheres.

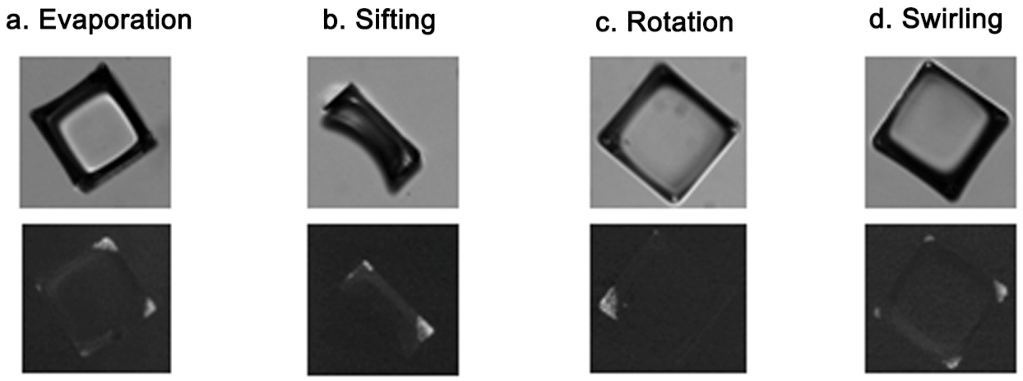

Figure S4. Representative brightfield microscopy images of MPS EMs that have been reformatted from a planar to a solution microarray using magnetic manipulation. Brightfield (top) and fluorescence (bottom) microscopy images show MPS EMs with different microparticle deposition methods, a) evaporation, b) sifting, c) rotation, and d) swirling. Microstructures are 100 μm on a side and are submerged in a liquid contained by a flat-bottomed vessel.

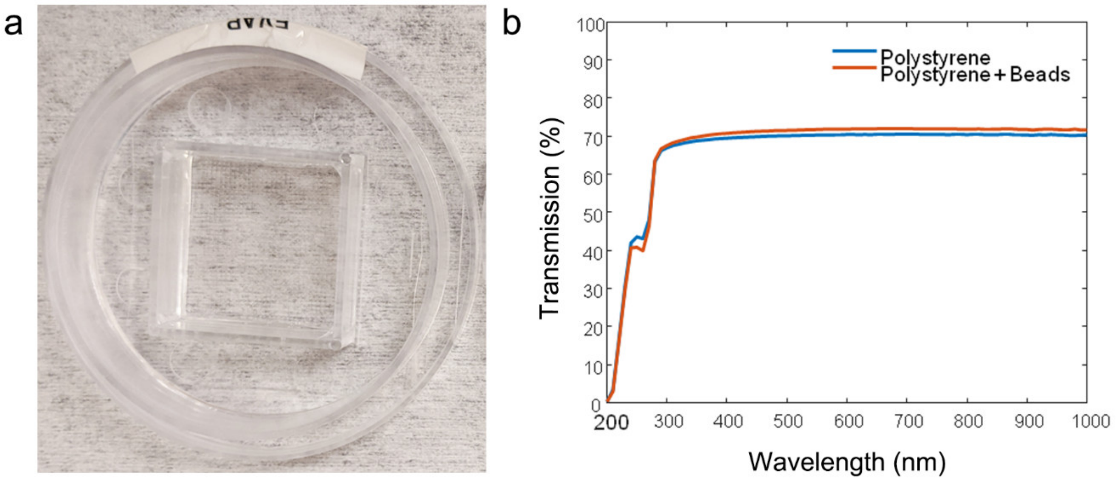

Figure S5. Characterization of optical transparency of MPS EMAs. (**a**) Digital photograph of an MPS EMA (central square) fabricated by the evaporation method and fitted to a culture chamber cassette. The texture of the fiber cloth can be seen through the MPS EMA. (**b**) Spectral transmission of EMAs with and without magnetic microbeads over UV-visible wavelengths.

Supporting References

1. DiSalvo, M.; Harris, D.M.; Kantesaria, S.; Peña, A.N.; Allbritton-King, J.D.; Cole, J.H.; Allbritton, N.L. Characterization of Tensioned PDMS Membranes for Imaging Cytometry on Microraft Arrays. Analytical Chemistry 2018, 90, 4792–4800, doi:10.1021/acs.analchem.8b00176.
2. Labelle, C.A.; Zhang, R.J.; Armistead, P.M.; Allbritton, N.L. Assay and Isolation of Single Proliferating CD4+ Lymphocytes Using an Automated Microraft Array Platform. IEEE Transactions on Biomedical Engineering 2020, 67, 2166–2175, doi:10.1109/TBME.2019.2956081.
3. Attayek, P.J.; Waugh, J.P.; Hunsucker, S.A.; Grayeski, P.J.; Sims, C.E.; Armistead, P.M.; Allbritton, N.L. Automated Microraft Platform to Identify and Collect Non-Adherent Cells Successfully Gene-Edited with CRISPR-Cas9. Biosens Bioelectron 2017, 91, 175–182, doi:10.1016/j.bios.2016.12.019.
